# The effects of multi-domain versus single-domain cognitive training in non-demented older people: a randomized controlled trial

**DOI:** 10.1186/1741-7015-10-30

**Published:** 2012-03-27

**Authors:** Yan Cheng, Wenyuan Wu, Wei Feng, Jiaqi Wang, You Chen, Yuan Shen, Qingwei Li, Xu Zhang, Chunbo Li

**Affiliations:** 1 Department of Psychiatry, Tongji Hospital, Tongji University School of Medicine, Shanghai 200065, China; 2 Department of Mental Health Centre, Yangpu District, Shanghai 200090, China; 3 Department of Biological Psychiatry, Shanghai Mental Health Centre, Shanghai Jiao Tong University School of Medicine, Shanghai 200030, China

## Abstract

**Background:**

Whether healthy older people can benefit from cognitive training (CogTr) remains controversial. This study explored the benefits of CogTr in community dwelling, healthy, older adults and compared the effects of single-domain with multi-domain CogTr interventions.

**Methods:**

A randomized, controlled, 3-month trial of CogTr with double-blind assessments at baseline and immediate, 6-month and 12-month follow-up after training completion was conducted. A total of 270 healthy Chinese older people, 65 to 75 years old, were recruited from the Ganquan-area community in Shanghai. Participants were randomly assigned to three groups: multi-domain CogTr, single-domain CogTr, and a wait-list control group. Twenty-four sessions of CogTr were administrated to the intervention groups over a three-month period. Six months later, three booster training sessions were offered to 60% of the initial training participants. The Repeatable Battery for the Assessment of Neuropsychological Status (RBANS, Form A), the Color Word Stroop test (CWST), the Visual Reasoning test and the Trail Making test (TMT) were used to assess cognitive function.

**Results:**

Multi-domain CogTr produced statistically significant training effects on RBANS, visual reasoning, and immediate and delayed memory, while single-domain CogTr showed training effects on RBANS, visual reasoning, word interference, and visuospatial/constructional score (all *P *< 0.05). At the 12-month posttest, the multi-domain CogTr showed training effects on RBANS, delayed memory and visual reasoning, while single-domain CogTr only showed effects on word interference. Booster training resulted in effects on RBANS, visual reasoning, time of trail making test, and visuospatial/constructional index score.

**Conclusions:**

Cognitive training can improve memory, visual reasoning, visuospatial construction, attention and neuropsychological status in community-living older people and can help maintain their functioning over time. Multi-domain CogTr enhanced memory proficiency, while single-domain CogTr augmented visuospatial/constructional and attention abilities. Multi-domain CogTr had more advantages in training effect maintenance.

**Clinical Trial Registration:**

Chinese Clinical Trial Registry. Registration number: ChiCTR-TRC-09000732.

## Background

Declining cognition in older people accounts for a major portion of increased health care costs [1]. As adults age, their risk for cognitive decline rises, resulting in an increasingly poor quality of life. Nearly half of community-living residents aged 60 years and over present to health care centers because of concerns about cognitive decline [2]. By 2050, the number of individuals over 65 years of age will increase to 1.1 billion worldwide, and as a result, the number of patients with dementia is estimated to reach 37 million [3].

High levels of mental activity can potentially decrease an individual's risk for developing dementia by approximately 50% [4] and can reduce the probability of incipient cognitive decline [5]. Valenzuela and colleagues have demonstrated a dose-response relationship between declining dementia risk and extent of mental activity in the later years of life [4]. Increasing mental activity in older people by directed cognitive training (CogTr) interventions represents a promising and novel approach to resisting age-related cognitive decline and even potentially curtailing the rise of dementia [6].

Several studies have explored the effects of CogTr interventions in healthy older individuals [7]. However, two recent meta-analyses drew different conclusions on whether cognitively intact older adults benefit from CogTr [7,8]. For the first time, Valenzuela *et al. *[8] focused on meta-analysis of the effect of CogTr on longitudinal performance in healthy adults. They demonstrated that CogTr could help slow the rate of age-related cognitive decline in a range of cognitive tasks with, on average, a moderate effect size (ES) of 0.6. They concluded that cognitive exercise training in healthy older individuals produces strong and persistent protective effects on longitudinal neuropsychological performance. On the other hand, Papp *et al. *[7] found no evidence that structured cognitive intervention programs delay or slow progression to Alzheimer's Disease (AD) in healthy older people. Because different clinical trials were identified within each review, a small, heterogeneous, and methodologically limited literature may be the reason for this negative conclusion. Among the studies included in the meta-analyses, most used single-domain CogTr, focusing either on memory [2,9-14], reasoning [2,15-17], processing speed [2,17-21], reading [22], solving arithmetic problems [22], or strategy training [23]. For example, the Advanced Cognitive Training for Independent and Vital Elderly trial (ACTIVE), the largest randomized control trial of single-domain interventions, explored how memory, reasoning, and processing speed training could improve targeted cognitive abilities. The results showed that CogTr enhances the targeted cognitive functions even in the 12-month follow-up evaluations [2]. Because single-domain CogTr intervention targets highly specific cognitive abilities, this type of intervention allows researchers to evaluate training-related effects on near-transfer measures [24]. However, single-domain CogTr neglects the complicated interactions between multiple mental processes required to create and preserve a viable and healthy mental state capable of the flexible thinking necessary to interact appropriately with one's world [24].

Previously, few studies evaluated the effects of multi-domain CogTr interventions in healthy older adults. Recently, Oswald *et al. *[25] demonstrated that multi-domain training (including memory, information processing, attention training) in 375 healthy older adults produced positive effects on cognition. Neely [26] conducted multi-domain training (including attention training, coding training and relaxation training) on community older residents and found a positive effect even at six-month follow-up.

The first aim of the present study was to explore whether community dwelling, healthy, older adults could benefit from CogTr. The second aim was to distinguish the different effects of single-domain and multi-domain CogTr interventions in healthy older adults. Specifically, we used a block-randomized, controlled, single blind trial to answer the following questions: Can healthy older adults benefit from CogTr? Does multi-domain CogTr improve and maintain cognitive abilities more than single domain CogTr? Can single-domain CogTr enhance untargeted cognitive functions in healthy older adults? Does booster training either augment or help maintain previous training effects? Which cognitive domain benefits most from CogTr?

## Methods

This study was approved by the Human Research Ethics Board of Tongji Hospital in Shanghai, China and all participants gave written informed consent before being enrolled in the study (LL(H)-09-04).

### Participants

All participants were community dwelling, older adults with good functional capacity and no evidence of significant cognitive impairment, living independently at the time of enrollment. The participants were recruited from three community centers around Tongji Hospital in Shanghai via a dispatched notice and broadcasting by the local institute of community service from March 2008 to April 2008.

All participants were interviewed in person by professional interviewers before being admitted to the study. To be eligible, subjects could not exhibit hearing, vision, or communication difficulties that would prevent completion of cognitive training. Vision, hearing and communication status were evaluated during the eligibility screening by a trained interviewer. Other eligibility criteria included age (65 ≤ age ≤ 75 years) at screening, and educational level (≥ 1 year). The Chinese version of the Mini-Mental State Examination score of 19 or above was required for enrollment [27]. The normal cut-off point of the MMSE is lower in China than in US due to a lower educational level [27]. Medical eligibility was assessed with a health status checklist designed for our study. Exclusion criteria included obvious cognitive decline, a diagnosis of AD, serious functional decline (having difficulty with independent living), and major medical or psychiatric conditions such as cancer, current chemotherapy or radiation treatment, major depression disorder, and schizophrenia).

### Study design

A block-randomized, controlled design was used to test whether community dwelling elderly could benefit from CogTr. Using the Statistics Analysis System (SAS) software, the randomization table was generated with a block randomization procedure (randomization occurred within three blocks) provided by an independent statistician who had no information about the study subjects. Each subject received a number within a concealed envelope indicating his/her randomization assignment. The study employed a three-group design, including one wait-list control group and two intervention groups, one receiving single-domain CogTr and one receiving multi-domain CogTr. According to the sample size equation n = (σ/Δ)^2^×(Ζ_α/2_+Ζ_β_)^2^, α = 0.05, Ζ_α/2 _= 1.96, β = 0.1, Ζ_β _= 1.65, the values of σ and Δ were determined on the basis of our previous studies. Combined with our previous drop-out rate, ninety individuals were assigned to each group. The statistical power level was set to 0.80, and the statistical significance level was set at 0.05. The non-training control group served to match for the social contact associated with CogTr and to determine the net effects on cognitive function specific to our CogTr intervention. Each intervention group received 24 sessions of CogTr over a three month period, conducted by qualified trainers. Three booster-training sessions were offered to 60% of the initial training sample six months after completion of the first 24 sessions of CogTr. All participants, including the control group, attended a lecture on different aspects of healthy living every two months. Cognitive assessors were blinded to intervention assignment. Training exposure and social contact were equal across interventions. Measurement outcomes were collected at baseline, immediately after CogTr completion, and during a 6-month and 12-month post training follow-up interview.

### Interventions

CogTr employs standardized exercises to provide structured practice of tasks relevant to specific aspects of cognition with the intention of specifically addressing cognitive functioning and/or cognitive impairment [28]. Most definitions of CogTr include four components: repeated practice, focusing on tasks that require problem solving, using standardized tasks, and targeting specific cognitive domains [6]. The CogTr interventions adopted for this study were based on the above definitions.

CogTr intervention took place in small group settings, with an average class size of 15 individuals. The training occurred at a frequency of twice a week over a 12 week period for 24 sessions from June to August 2009. The multi-domain CogTr targeted on memory, reasoning, problem solving strategies, visuospatial map reading skill development, handcraft making, and health and physical exercise. The single-domain CogTr focused specifically on reasoning training, including the towers of Hanoi, numerical reasoning, Raven Progressive Matrices, and verbal reasoning.

Each session lasted 60 minutes. A lecture was presented during the first 15 minutes of each hour, focusing on education about the diseases common in older persons. Then, all participants were trained in one specific cognitive technique during the second 30 minutes of each session. The trainer taught the participants about a certain strategy or technique. All participants received instructions about the rule, including its methods and function and how to use it in daily life, such as Loci memory training, face/name memory training, and so on. Sometimes, the participants needed to be tested on the content to assess their learning success. The last 15 minutes were used to consolidate the newly practiced skills by solving some real-life problems. Participants received structured training in topics delivered via PowerPoint presentations. The instructor followed a manual of structured curricular format for training, but allowed discussion based on the needs and interests of participants. Participants were assigned homework once a week including cognitive tasks relating to the content of the latest session, health knowledge reading, Chinese calligraphy, and simple graph drawing. The homework was reviewed every week.

At the end of each session, participants evaluated the type of intervention used for that session. Subjects were asked to provide feedback on performance achievement, to describe their opinion of or to provide suggestions for each intervention, and to document the self-training practice they performed outside the training sessions during their daily life in a booklet which they handed in to the study personnel after completing all 24 training sessions. A study observer monitored each individual's performance and assessed the quality of the skill-related practice during the intervention. The observers provided useful feedback to the instructor with the aim of preventing participants from developing poor practice habits and precluding the inadvertent reinforcement of detrimental training.

Six months after completion of the initial 24 cognitive training sessions, booster training was provided to a randomly selected 60% of subjects in each intervention group. Booster training, which comprised one additional 60-minute training session every month for the next three months from April to June 2010, reinforced the initial training by reviewing previously practiced content.

### Measures

Composite outcome measures were created to represent multiple cognitive domains that matched the content of each intervention. All measures were administered at baseline and three times post-test after the intervention. Each of the composite measures was designed to assess ability rather than performance on a single test. The outcome measures included the Repeatable Battery for the Assessment of Neuropsychological Status (RBANS, Form A) which has good reliability and validity in a Chinese community-living older sample [29,30], the Color Word Stroop test (CWST), the visual reasoning test and the trail making test (TMT). These measures were chosen for their sensitivity to the cognitive functions most vulnerable to aging. Details of each test are given below.

### The RBANS

The RBANS consists of six scores: Total Score and five Index Scores. The latter include Immediate Memory, Visuospatial/Constructional, Language, Attention, and Delayed Memory. With the exception of the delayed memory index which is based on four subtests, each of the other four Index Scores is based on two subtests. The RBANS was designed for evaluation of disease progression and intervention efficacy [31]. All participants in the present study received a version of form A of the RBANS modified for Chinese subjects without altering difficulty level. For example, 'Fujian, China' was substituted for 'Cleveland, Ohio' in the story memory and story recall test. Administration and scoring of the RBANS were conducted by trained study personnel according to the directions detailed in the manual [31].

### The CWST

The Color Word Stroop Test (CWST) was administered according to standard techniques [32]. The most common CWST indices include the number of naming errors and reaction time. Interference scores are measured by both color-interference time, defined as the time difference required for accurately reading aloud color-ink words versus black-ink words, and word-interference time, the delay in identifying the ink-color of color words versus non-color words [33]. The CWST was used to track perseverations, a measure of executive functioning, attention capacity across different experimental conditions and reading speed [34,35].

### The Visual Reasoning Test

The visual reasoning test is a part of the visual gnostic function tests included in the the World Health Organization Neuropsychological Battery of Cognitive Assessment Instruments for the elderly (WHO-BCAI). It consists of nine items. The test involves tasks requiring identification of patterns in graphic series problems. The visual reasoning test is correlated with cognitive functions of reasoning, summarizing, and analytical ability [36].

### The Trail Making Test

The Trail Making Test (TMT) is one of the most frequently used neuropsychological tests in clinical practice, largely due to its good reliability, validity, and sensitivity to cognitive impairment and brain damage [37]. The TMT is a pencil and paper task, requiring participants to connect a series of circles in order. Part A of the test (Trails A) includes numbered circles only, whereas Part B (Trails B) contains numbered and colored circles and necessitates alternating between number and color to correctly connect each circle [38]. Successful completion of both trails depends on visual scanning, motor speed and dexterity, speeded processing, while trail B also requires flexibility, working memory, and alternating attention [38]. We noted completion time, number of errors, number of reminder prompts and number of approximate errors for both trails. The approximate errors referred to errors found by the participants and revised before they finished the test.

### Statistical analysis

To evaluate the effects of initial CogTr and booster training over one year, a general linear model (GLM) - repeated measures was used. The within-subject factor was defined as time, and the between-subject factor was defined as group. The within-subject dependent variables were outcome measures at four time points: baseline, immediately after intervention completion, 6 months post-test and 12 months post-test. The outcome measures at baseline were defined as covariates to control for the slight, but not statistically significant, between group imbalance prior to intervention. Main effects for group and time were determined. The time*group interaction determined the net effect of CogTr intervention, whereas the time*booster*training interaction assessed the training-specific effects of each booster intervention [2]. The repeated measures pairwise comparisons were Bonferroni corrected to protect against false positives [9]. The intention-to-treat analysis (ITT) was used to assess CogTr effects. Those participants who attended at least one of the CogTr sessions and were followed up after intervention were all included in the statistical analysis [2].

The net effect size (NES) was used to compare outcome scores (immediate, 6-month, and 12-month post training) to baseline scores and to control group scores. The NES of training at a specific timepoint was defined as: ((trained mean - control mean at timepoint n) - (trained mean - control mean at baseline))/intra-subject SD. Similarly, the NES of each booster training was defined as: ((booster mean - non-boosted mean at timepoint n) - (booster mean - non-booster mean at baseline))/I ntra-subject SD [2]. In addition, covariate-adjusted intervention effects were examined by including covariates of outcome measures at baseline in all analyses. Analyses were performed using the SPSS, version 17.0, with *P *< 0.05 as the significance level.

## Results

### Demographic information

**Figure 1:**
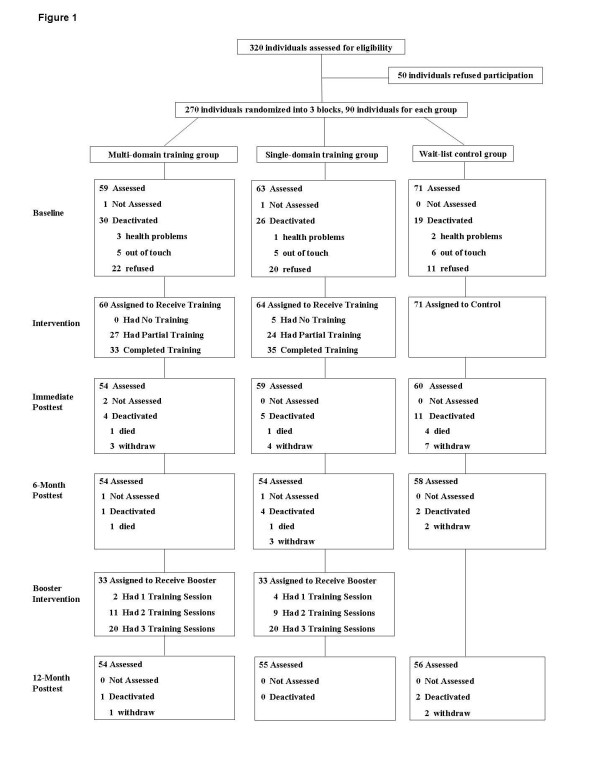
**Flow of participants through the trial**. 'Refused' due to lack of interest in continuing, repeatedly missed appointments, scheduling conflicts. 'Not Assessed' due to temporary health problems or scheduling conflicts, but willing to keep participating in our research. 'Out of touch' due to home phone or address change, stay out. 'Died' includes cancer, stroke, cardiac sudden death. 'Withdraw' indicates subjects withdrew for reasons including the death of a family member, poor health, lack of interest, had no time, changed contact, stressed. 'Completed Training' means subjects complete 80% of the training sessions (≥ 19 training sessions).

From the 320 eligible individuals who were contacted for participation, 270 agreed to attend our study while 50 subjects refused to participate prior to randomization from March 2008 to April 2008. Reasons for refusal included lack of enthusiasm, too busy to participate, and unwillingness to attend the training sessions. Ninety individuals were randomly assigned to each group. One hundred and ninety three individuals completed cognitive assessments at baseline from November to December in 2008, while 173 subjects finished the immediate follow-up after CogTr from September to October in 2009, 166 subjects finished the six-month follow-up after CogTr from March to April in 2010 and 165 subjects finished the one-year follow-up after CogTr from October to November in 2010, respectively. The flow of participants through the entire study is illustrated in Figure 1.

The intervention groups were comparable with the control group with regard to age, education and sex composition at each assessment. Subject characteristics of the study groups are summarized in Table 1.

**Table 1:** Characteristics and comparability of our trial

		Multi-domain training	Single-domain training	Control group	F/X2	*P*
Age	Randomization	70.79 ± 3.33	69.78 ± 3.76	70.17 ± 3.47	1.88	0.15
($\bar{\text{X}}$ ± SD)	Baseline	70.76 ± 3.49	69.79 ± 3.93	70.27 ± 3.41	1.10	0.34
	Immediate Posttest	71.61 ± 3.70	70.42 ± 3.87	71.10 ± 3.84	1.39	0.25
	6-Month Posttest	72.46 ± 3.69	71.35 ± 3.96	72.09 ± 3.72	1.20	0.30
	12-Month Posttest	72.78 ± 3.62	71.51 ± 4.11	72.39 ± 3.77	1.57	0.21
Education^a^	Randomization	9.33 ± 3.80	9.77 ± 3.96	9.58 ± 3.93	0.28	0.76
($\bar{\text{X}}$ ± SD)	Baseline	10.15 ± 3.57	9.16 ± 4.00	9.08 ± 4.13	1.44	0.24
	Immediate Posttest	9.65 ± 4.49	9.02 ± 4.07	8.94 ± 4.20	0.47	0.63
	6-Month Posttest	9.80 ± 4.43	8.93 ± 4.54	8.52 ± 4.10	1.25	0.29
	12-Month Posttest	9.96 ± 4.18	9.30 ± 4.23	8.50 ± 3.91	1.75	0.18
Sex composition	Randomization	52:38	41:49	45:45	2.76	0.25
(male: female)	Baseline	36:23	26:37	37:34	4.79	0.09
	Immediate Posttest	33:21	26:33	31:29	3.29	0.19
	6-Month Posttest	33:21	22:32	30:28	4.49	0.11
	12-Month Posttest	33:21	23:32	30:26	4.14	0.13

Note: ^a^Education refers to self-reported years of education

### Intervention compliance

**Figure 2:**
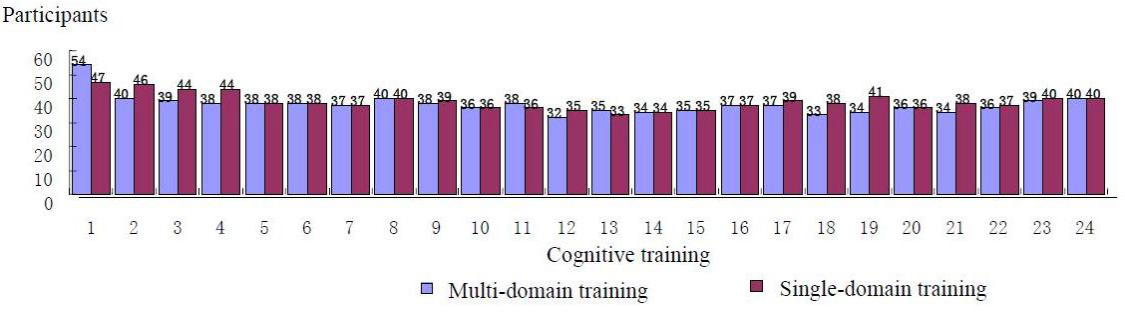
**Attendance of each session**.

Intention-to-treat analyses were used. Sixty individuals attended at least one session of multi-domain CogTr, while 59 individuals completed a single-domain CogTr session (detailed in Figure 1). The percentage of participants who were considered study completers (attended ≥ 19 sessions) was not significantly different between multi- and single-domain CogTr (55% and 54.69%, respectively (*P *> 0.05)). Attendance-rates per session for the two intervention groups were also not statistically different (63.35% and 61.38%, respectively, (X^2 ^= 1.21, *P *> 0.05)). Attendance data for each session is detailed in Figure 2.

### Cognitive outcomes at baseline

Cognitive performance outcome measures at baseline are summarized in Table 2. No group differences were recorded at baseline in almost all measures except for the approximate error number of TMT trails A (F = 4.31, *P *= 0.02), indicating that cognitive abilities across study groups were largely comparable before training. The Students-Newman-Keuls test was used to draw a parallel between groups. We found that although the two intervention groups showed no significant between group baseline differences, the intervention groups (M = 0.02) together were significantly different from the control group (M = 0.11) at baseline. The control group showed a little more approximate error number.

**Table 2:** Cognitive Outcomes at Baseline

	A (Mean ± SD)	B (Mean ± SD)	C (Mean ± SD)	F
RBANS total score	86.05 ± 14.11	85.95 ± 14.17	83.34 ± 14.54	0.78
Immediate memory	80.81 ± 15.60	79.84 ± 15.51	77.15 ± 15.21	1.00
Visuospatial/Constructional	97.75 ± 16.37	93.87 ± 16.24	94.44 ± 16.50	1.00
Language	94.05 ± 11.64	92.76 ± 9.66	94.32 ± 8.70	0.45
Attention	84.90 ± 17.37	87.54 ± 16.86	83.38 ± 16.37	1.03
Delayed Memory	89.80 ± 17.57	93.00 ± 17.42	86.76 ± 17.36	2.14
The Visual Reasoning Test	4.63 ± 1.86	4.92 ± 1.90	4.70 ± 2.21	0.35
The CWST				
Color interfere	20.98 ± 14.99	17.26 ± 9.50	20.97 ± 12.71	1.84
Word interfere	44.76 ± 25.53	39.95 ± 17.42	41.15 ± 19.57	0.85
Number of naming errors	10.67 ± 11.02	8.39 ± 7.58	11.68 ± 12.59	1.61
The TMT				
Trails A complete time	113.25 ± 50.30	107.55 ± 55.93	114.52 ± 51.89	0.32
Trails A error number	0.47 ± 0.90	0.48 ± 1.08	0.51 ± 1.01	0.02
Trails A reminding time	0.02 ± 0.13	0.03 ± 0.25	0.06 ± 0.23	0.56
Trails A approximate error number	0.02 ± 0.13	0.02 ± 0.13	0.11 ± 0.32	4.31*
Trails B complete time	205.64 ± 114.43	201.15 ± 101.97	222.96 ± 124.15	0.68
Trails B error number	0.64 ± 1.87	0.69 ± 1.98	0.77 ± 1.94	0.07
Trails B reminding time	0.36 ± 1.40	0.23 ± 1.08	0.41 ± 0.98	0.40
Trails B approximate error number	0.19 ± 0.44	0.11 ± 0.45	0.09 ± 0.28	1.12
MMSE	27.16 ± 2.13	27.33 ± 2.14	26.86 ± 2.22	0.79

* *P *< 0.05; A: Multi-domain Training Group; B: Single-domain Training Group; C: Control Group. CWST, Color Word Stroop test; MMSE, Mini-mental State Examination; RBANS, Repeatable Battery for the Assessment of Neuropsychological Status; TMT, Trail Making test.

### Effect of CogTr and booster training

**Figure 3:**
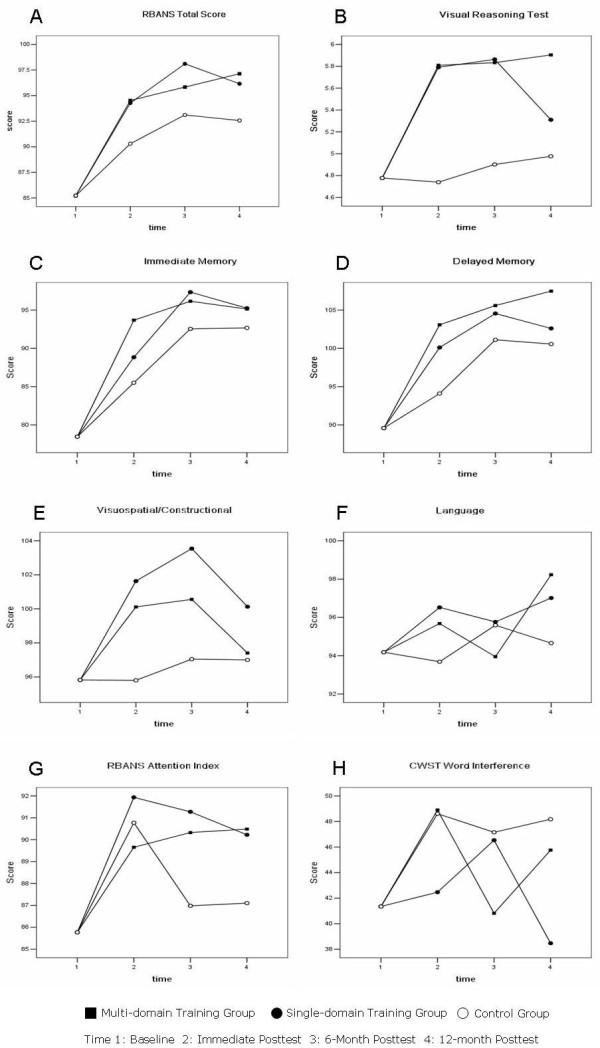
**Temporal trends of measure outcomes for each group**. (**A**) RBANS total score. (**B**) Visual reasoning test score. (**C**) RBANS immediate memory index score. (**D**) RBANS delayed memory index score. **(E**) RBANS visuospatial/constructional index score. (**F**) RBANS language index score. (**G**) RBANS attention index score. (H) CWST word interference score. CWST, Color Word Stroop Test; RBANS, Repeatable Battery for the Assessment of Neuropsychological Status;.

The results of the GLM-repeated measures estimations are summarized in Table 3 Table 4 and Figure 3. Table 3 shows the main effect of time and group and interaction for time*group. To compare the effect of intervention groups from baseline to each posttest, the NES for each outcome measure is listed in Table 4. The measure outcomes which showed no significant effect of group or group*time are not listed in the results of pairwise comparison for groups in Table 4. Temporal trends of measure outcomes for each group are presented in Figure 3.

**Table 3:** Cognitive Training Effect

	Time	Group	Time*Group
RBANS total score	11.70***	6.05**	3.05**
Immediate memory	21.75***	2.67	2.22*
Visuospatial/Constructional	35.53***	4.02*	1.96
Language	41.09***	1.34	2.40*
Attention	15.81***	1.39	1.36
Delayed Memory	27.00***	6.09**	3.06**
The Visual Reasoning Test	26.86***	8.07***	3.44**
The CWST			
Color interference score	21.92***	0.04	0.83
Word interference score	14.21***	1.47	2.02
Number of naming errors	4.46**	0.62	0.38
The TMT			
Trails A complete time	10.47***	0.79	0.68
Trails B complete time	20.79***	0.13	0.42
Trails A approximate error number	6.04***	0.01	0.39
MMSE	17.33***	0.30	0.62

* *P *< 0.05; ** *P *< 0.01; *** *P *< 0.001. CWST, Color Word Stroop test; MMSE, Mini-mental State Examination; RBANS, Repeatable Battery for the Assessment of Neuropsychological Status; TMT, Trail Making test.

**Table 4:** Net effect of training group^a^

	Multi-domain Training	Single-domain Training
	
	Net Effect Size(*P *value)	Net Effect Size(*P *value)
RBANS Total Score		
Immediate Posttest	0.296(0.017)	0.279(0.023)
6-Month Posttest	0.191	0.350(0.006)
12-Month Posttest	0.320(0.026)	0.252
Visual Reasoning Test		
Immediate Posttest	0.533(0.006)	0.524(0.006)
6-Month Posttest	0.465(0.016)	0.479(0.011)
12-Month Posttest	0.463(0.012)	0.138
RBANS Immediate Memory		
Immediate Posttest	0.530(0.002)	0.216
6-Month Posttest	0.234	0.311
12-Month Posttest	0.160	0.167
RBANS Delayed Memory		
Immediate Posttest	0.511(0.000)	0.343(0.029)
6-Month Posttest	0.255	0.196
12-Month Posttest	0.394(0.011)	0.116
RBANS Visuospatial/Constructional		
Immediate Posttest	0.263	0.356(0.031)
6-Month Posttest	0.215	0.397(0.014)
12-Month Posttest	0.025	0.191
CWST (word interfere)		
Immediate Posttest	0.014	-0.294
6-Month Posttest	-0.303	-0.030
12-Month Posttest	-0.116	-0.464(0.048)

^a^Only significant *P *values are reported. CWST, Color Word Stroop test; RBANS, Repeatable Battery for the Assessment of Neuropsychological Status.

### General cognitive abilities

RBANS total score revealed a significant main effect of time, group, and significant interaction between time and group (all *P *values < 0.01). The NES for each intervention group showed a significantly improved effect. The immediate posttest NES showed no significant difference between multi-domain training and single-domain training (d = 0.027, *P *> 0.05). Single-domain CogTr showed a better effect at the 6-month posttest, while multi-domain CogTr resulted in a better effect at the 12-month posttest. The multi-domain CogTr group presented a stable increase from the pretest to each posttest evaluation (F = 5.873, *P *= 0.001), whereas it remained stable at 12-month posttest (d _time 4-2 _= 2.680). In contrast, the performance of the single-domain CogTr group decreased from 6-month posttest to 12-month posttest (d _time 4-3 _= -1.981) (Figure 3A). The main effect of booster training showed a significant booster effect (d = 1.808, F = 3.991, *P *= 0.048), without a significant difference between groups (F = 0.457, *P *= 0.500).

The Mini-mental State Examination (MMSE) score revealed no significance of either a group main effect or time*group interaction, whereas the main effect of time was significant (F = 17.33, *P *< 0.001). The effect of booster training showed no significant difference among groups (F = 1.108, *P *= 0.294).

### Reasoning abilities

The visual reasoning test score revealed a significant main effect of time, group, and interaction between time and group (all *P *values < 0.01). Then NES for each intervention group showed a significantly improved effect after training, especially at the immediate posttest. There was no significant difference in the NES between multi-domain and single-domain CogTr at the immediate (d = 0.009, *P *> 0.05) or six-month posttest (d = -0.014, *P *> 0.05). The multi-domain CogTr group gradually increased from pretest to each posttest evaluation (F = 5.774, *P *= 0.001), whereas it remained stable and at a good effect level at the 12-month posttest. In contrast, the performance of the single-domain CogTr group decreased from 6-month posttest to 12-month posttest (d _time 4-3 _= -0.538) and led to a small effect at 12-month posttest (Figure 3B). The performance of the booster participants showed a significant booster effect for reasoning compared to the non-booster participants (F = 20.700, *P *= 0.000). Single-domain booster training (NES = 0.863, *P *< 0.001) showed a better booster effect than multi-domain booster training (NES = 0.532, *P *= 0.024).

### Memory ability

RBANS immediate memory index score revealed a non-significant main effect of group but significant main effect of time and a significant interaction between time and group. The NES for multi-domain CogTr showed a significantly improved immediate effect after intervention, which is better than the single-domain CogTr performance. The NES revealed scores increased from the pretest to each posttest evaluation (Figure 3C). Booster training showed no significant booster effect (F = 1.640, *P *= 0.202).

RBANS delayed memory index score revealed a significant main effect of time, group, and interaction between time and group (all *P *values < 0.01). The NES for multi-domain CogTr showed a significantly improved immediate effect after training, a stronger effect than single-domain CogTr performance. Furthermore, the multi-domain CogTr effect remained stable at the 12-month posttest. The temporal trend showed the multi-domain CogTr group score as a steadily increasing line from the pretest to each posttest evaluation. In contrast, the performance of the single-domain CogTr group decreased from the 6-month posttest to the 12-month posttest (d _time 4-3 _= -1.717) (Figure 3D). Booster training resulted in no significant booster effect between groups (F = 3.598, *P *= 0.060).

### Visuospatial/Constructional abilities

The RBANS visuospatial/constructional index score revealed significant main effects of time and group, whereas their interaction showed a non-significant effect. The NES for single-domain CogTr showed significant improvement after intervention, which showed a better effect than the multi-domain CogTr group at all posttest performance. Temporal trends of intervention groups showed a similar performance, a gradual increase after training and a decrease from the 6-month to 12-month posttest (Figure 3E). The performance of the booster participants indicated a significant booster effect (F = 4.489, *P *= 0.036) compared to the participants who did not attend a booster session (F = 0.447, *P *= 0.505).

### Language abilities

The RBANS language index score revealed a significant main effect of time and interaction between time and group but a non-significant main effect of group. Time differences showed that multi-domain CogTr resulted in a significant increase at 12-month posttest (d_time4- 1 _= 3.740, *P *= 0.008), while single-domain CogTr increased significantly at immediate (d_time2-1 _= 2.679, *P *= 0.046) and 12-month posttest (d_time4-1 _= 3.208, *P *= 0.029). Booster training showed no significant booster effect (F = 1.284, *P *= 0.259).

### Attention abilities

The RBANS attention index score only showed a significant main effect of time but no effect of group or interaction. Temporal trends indicated that the single-domain CogTr group improved more than the multi-domain CogTr group after intervention (Figure 3G). Booster training resulted in no significant booster effect (F = 2.408, *P *= 0.123).

The CWST interference score and number of naming errors showed a significant effect of time but a non-significant effect of group and interaction. The word interference score of the single-domain CogTr group showed a significant training effect at the 12-month posttest. Booster training had no significant effect on the CWST interference score or the number of naming errors.

### Speeded Processing Function

The completion time of Trails A and B, and the approximate error number of Trail A of the TMT showed a significant effect of time but no effect of group or interaction. Booster training had a significant effect on completion time (F _trails A _= 6.289, *P *= 0.013, F _trails B _= 7.352, *P *= 0.007).

### Impact of completed training sessions on cognitive outcomes

The attendance-rates per session for the two intervention groups were not statistically different. To explore the impact of completed training sessions on cognition, we divided the training subjects into two groups according to whether they had completed 80% of the training sessions (≥ 19 sessions): completed training group and partial training group. Controlling the baseline assessment as a covariate, we compared group differences at the three time-points of every cognitive test. There were ten posttest assessments that showed group differences (*P *< 0.05); the completed training group outperformed the partial training group. Detailed data are shown in Table 5.

**Table 5:** Impact of Completed Training Sessions on Cognitive Outcomes

	Immediate Posttest			6-month Posttest			12-month Posttest		
	
	A($\bar{X}$)	B($\bar{X}$)	F	A($\bar{X}$)	B($\bar{X}$)	F	A($\bar{X}$)	B($\bar{X}$)	F
Visuospatial/Constructional	104.00	98.06	6.85*	105.80	98.42	10.01**	101.48	97.07	3.84
Visual Reasoning Test	6.10	5.65	1.89	6.26	5.37	7.03**	6.14	5.06	11.27**
The CWST									
Card C Missing Num.	-0.04	0.65	5.02*	0.07	0.12	0.38	-0.01	0.25	2.26
Card D Correct Num.	46.11	43.31	8.52**	46.30	45.62	0.59	46.17	44.52	2.16
Card D Error Num.	3.78	6.42	7.63**	3.52	4.28	0.78	3.80	5.49	2.28
The TMT									
Trails A Errors Num.	0.26	0.32	0.17	0.26	0.52	2.86	0.18	0.64	6.38*
Trails A Prompts Num.	0.13	0.32	3.38	-0.00	0.27	9.63**	0.15	0.08	0.48
Trails B Completion T.	174.30	216.23	5.44*	180.65	206.56	2.15	172.96	176.29	0.07
Trails B Errors Num.	0.30	0.33	0.02	0.19	0.92	8.19**	0.22	0.62	3.22
Trails B Prompts Num.	0.20	0.39	1.83	0.12	0.80	11.32**	0.37	0.43	0.15

* *P *< 0.05; ** *P *< 0.01; $\bar{\text{X}}$: Mean; A: completed training group; B: partial training group. CWST, Color Word Stroop test; TMT, Trail Making test.

## Discussion

To the best of our knowledge, this is the first randomized, controlled study to compare multi-domain with single-domain CogTr intervention in community-living healthy older people. Immediate and long-term follow-up provided powerful proof for the improvement and maintenance of training-related effects on cognitive function.

The data presented here demonstrated that: (1) cognitive function varied along with time. RBANS total score, index scores and the visual reasoning test showed that the change in cognitive outcomes varied with time due to different group design and that each group benefitted differently after CogTr. CogTr was effective in improving test-related cognitive functions. (2) Multi-domain CogTr produced a significant effect on the RBANS total score, the visual reasoning test and immediate and delayed memory indices, while single-domain CogTr showed improvement in RBANS total score, the visual reasoning test, delayed memory and visuospatial/constructional index score, and CWST word interference. These results demonstrated that both multi-domain and single-domain CogTr resulted in training-related effects in cognitive improvement. Multi-domain CogTr resulted in better improvement in memory ability, while single-domain training gave better results for visuospatial/constructional and attention ability. (3) Besides the visual reasoning test, single-domain CogTr revealed effective improvement on the RBANS total score, delayed memory, visuospatial/constructional abilities, language, and CWST word interference. These results showed the generalization of benefit to non-trained cognitive functions, and such benefits persist. (4) It is notable that the RBANS total score, delayed memory and the visual reasoning test showed significant training effects in the multi-domain CogTr group at the 12-month posttest, while the single-domain CogTr only showed training effect on CWST word interfere at the 12-month posttest. The multi-domain CogTr effect showed better performance than single-domain CogTr at long-term follow-up and resulted in a steady gradual increase after intervention in most measure outcomes. These results proved that multi-domain CogTr had a better training effect on maintenance. (5) Booster training had a significant effect on the RBANS total score, the visual reasoning test, the completion time of TMT, and the visuospatial/constructional index score. These results prove that booster training was effective and enhanced the initial training effect on reasoning, and executive and visuospatial/constructional ability. (6) Reasoning ability showed a good training effect in both intervention groups and had no significant group differences at immediate and 6-month follow-up. Meanwhile, the multi-domain group showed better performance at the 12-month follow-up, which was consistent with the training effect maintenance on other cognitive tests. Furthermore, booster training also had a significant effect on the visual reasoning test. These results indicate that reasoning ability may be the domain most sensitive to CogTr (7). The completed training group outperformed the partial training group on visuospatial/constructional, reasoning, attention and processing speed abilities. These results also proved that cognitive training was effective.

Compared to previous studies on CogTr in healthy older adults [2,8,25,39-42], our study drew some similar conclusions. First, CogTr in healthy older people produced positive effects. CogTr helped normal older people perform better on a series of measures of specific cognitive abilities. Effect sizes of cognitive abilities posttest are mostly consistent with previous research [2,8,25,39-42]. Second, CogTr can result in a generalization of the training effect since untargeted cognitive domains also showed a better performance after single-domain CogTr and the effect was maintained [2,37,38]. Beneficial effects of CogTr were limited not only to trained functions but extended to other cognitive abilities. Third, booster sessions consolidated the effects of initial training [2,37], although the effects were limited to reasoning, visuospatial/constructional abilities and faster processing.

Some important findings are described in this study for the first time. First of all, the comparison between multi-domain and single-domain CogTr with healthy older adults found that certain domains benefit differently from CogTr. Memory ability benefits more from multi-domain CogTr, while visuospatial/constructional and attention abilities benefit more from single-domain CogTr. Furthermore, although the CogTr impact on cognitive function decreased over time, it remained statistically significant, attesting to the durability of the intervention effects. Multi-domain CogTr has more advantages for effect maintenance. Secondly, the largest effect was observed in reasoning ability. The NES of initial training and booster training were larger than the values showed in the ACTIVE study [2]. The difference may be due to more training sessions (24 versus 10) in our study. Thus, we concluded that reasoning ability may be the domain that is most sensitive to CogTr.

It is interesting that the single domain training, which focused on reasoning, outperformed multi-domain in other cognitive domains but not in the reasoning domain. Why did single-domain training outperform multi-domain training in other cognitive domains? The reason might be the generalization of the training effect. Several studies have proved that non-trained cognitive domains could also be improved after single-domain training [43,44]. The reason why multi-domain training outperformed single domain training in the reasoning domain may be that reasoning, as an important mental process, needs to collaborate with other brain processes. Single-domain CogTr usually doesn't consider the elaborate collaboration with other mental processes, which is necessary to create and maintain a viable healthy mind capable of flexibility in thinking, recalling, linking, and reacting to one's world [24]. Thus, multi-domain training may have more advantages than single-domain training because of the collaboration.

One limitation of this study is that 53 subjects drop out before completing the baseline cognitive testing due to the following reasons. First, there was a long time lag between randomization and baseline assessment, therefore, some older adults had totally forgotten our study or changed their contact information. Second, the baseline assessment was conducted in summer. Some older adults refused to attend the assessment due to the hot weather. Third, all subjects in our study came from three communities in Shanghai, which could not be fully representative of the entire population of non-demented older people in China. This may cause bias in our study and limit the generalization of our results. We may need to select multiple cities for recruitment in the future to avoid this bias. Whether our conclusions could be applied to other populations deserves to be further studied. Although the limitations exist, this study represents an important extension of previous knowledge of the effects of CogTr on brain aging. Several cognitive domains, such as memory, visual reasoning, visuospatial/constructional and attention improved significantly after CogTr. More importantly, such benefits were maintained over time. The clinical role of CogTr with healthy individuals was primary prevention to reduce disease incidence [6]. Decline in cognitive abilities has been shown to lead to an increased risk of functional difficulty in independent living [39]. There was evidence that improvements in cognitive function can have a positive effect on daily functioning [39]. The generalization of the training effect on daily functioning has also been seen in several studies [45,46]^. ^Thus, CogTr not only can improve cognition but may also have positive effects on daily functioning. CogTr, which produces no toxic effects, deserves to be further studied for its possible preventive and palliative therapeutic value. With acceptable compliance and training benefits and reasonable cost of implementation, our study may encourage future use of these CogTr methods to improve or maintain cognitive and daily function in cognitively impaired older adults.

## Conclusions

Overall, cognitive training can improve memory, visual reasoning, visuospatial/construction, attention and neuropsychological status in community-living older persons and can help maintain their functioning over time. Multi-domain CogTr enhanced memory proficiency and single-domain CogTr augmented visuospatial/constructional and attention abilities, while multi-domain CogTr had more advantage in training effect maintenance. Booster training measurably enhances the training efficacy.

## Abbreviations

ACTIVE: Advanced Cognitive Training for Independent and Vital Elderly; AD: Alzheimer's Disease; CogTR: cognitive training; CWST: Color Word Stroop Test; ES: effective size; GLM: general linear model; ITT: intention to treat; MMSE: mini-mental state examination; NES: net effect size; RBANS: Repeatable Battery for the Assessment of Neuropsychological Status; TMT: trail making test.

## Competing interests

The authors declare that they have no competing interests.

## Authors' contributions

Y C carried out the cognitive training and booster training, drafted the manuscript and participated in the training design. WF, YC and YS contributed to the participants' enrollment and cognitive assessment. JQW helped carry out the training. QWL and XZ contributed to the data collection and performed the preliminary data preparation. CBL conceived of and designed the study and performed the randomization procedure, and reviewed the manuscript. WYW conceived of the study, and participated in its design and coordination and helped review the manuscript. All authors have read and approved the final manuscript.

## Pre-publication history

The pre-publication history for this paper can be accessed here:

http://www.biomedcentral.com/1741-7015/10/30/prepub
